# Supplementary material for: Prevention of dsRNA‐induced interferon signaling by AGO1x is linked to breast cancer cell proliferation
Source: EMBO J. 2020 Aug 19;39(18):e103922. doi: 10.15252/embj.2019103922 (PMC7507497; doi:10.15252/embj.2019103922)
Supplement: Supplementary file 1 — Appendix [file EMBJ-39-e103922-s001.pdf]

Appendix for "AGO1x prevents dsRNA-induced interferon signaling to promote proliferation of breast cancer cells"

Souvik Ghosh[1,7], Joao C Guimaraes[1,7,*], Manuela Lanzafame[2], Alexander Schmidt[3], Afzal Pasha Syed[1], Beatrice Dimitriades[1], Anastasiya Börsch[1], Shreemoyee Ghosh[1], Nitish Mittal[1], Thomas Montavon[6], Ana Luisa Correia[4], Johannes Danner[5], Gunter Meister[5], Luigi M. Terracciano[2], Sébastien Pfeffer[6], Salvatore Piscuoglio[2,4], and Mihaela Zavolan[1,*]

Table of Contents **Page No**

**1. Appendix Figure legends**	**2**
**2. Appendix Figure S1**	**6**
**3. Appendix Figure S2**	**7**
**4. Appendix Figure S3**	**8**
**5. Appendix Figure S4**	**9**
**6. Appendix Figure S5**	**10**
**7. Appendix Figure S6**	**11**
**8. Appendix Figure S7**	**12**
**9. Appendix Figure S8**	**13**
**10. Appendix Figure S9**	**14**
**11. Appendix Figure S10**	**15**
**12. Appendix Figure S11**	**16**
**13. Appendix References**	**17**

Appendix Figure legends

Appendix Fig S1 Expression profile of *AGO1* in normal tissues.

A, Expression level of *AGO1* transcript in tissues represented in the human protein atlas (Uhlén *et al*, 2015). The height of the bars represents the mean expression across multiple samples (n=1-13, depending on the tissue). Red dotted lines indicate +/- 2 standard deviations.

Appendix Fig S2 AGO1x detection in multiple cell types.

A, Western blot analysis of AGO1x expression in multiple mammalian cell extracts. RIPA buffer lysed extracts obtained from the indicated cell lines were blotted on nitrocellulose membrane and probed for AGO1x and Tubulin. **B,** Western blot analysis of AGO1x expression in wild type MDA-MB-231 and HEK 293T cells. Cells were transiently transfected with FLAG-tagged AGO1x constructs and lysates were used in the same blot. MDA-MB-231 expressing the FLAG-tagged AGO1 serves as a control for the specificity of the antibody and to validate the higher MW of the AGO1x protein relative to AGO1. **C**, qRT-PCR-based estimation of endogenous let-7a miRNA levels in the indicated cell lines. For comparison, U6 snRNA was used as invariant control. Bar plots show the abundance of let-7a in MDA-MB-231 and HeLa cells relative to the HEK293T in the same experimental series, the latter being represented as unit.

Appendix Fig S3 AGO1x localizes to the nucleus, in the vicinity of nucleoli.

A, Immunofluorescence imaging of HeLa cells co-stained with AGO1 (green) and AGO1x (red) antibody. DAPI was used to mark the nucleus (blue). Images were obtained with a Nikon Ti-E inverted microscope, cells were visualized with a CFI Plan Apochromat DM 60x lambda oil (NA 1.4) objective, and images were captured with a Hamatsu Orca-Flash 4.0 CMOS camera. **B**, Representative stacks of a MDA-MB-231 cell nucleus. The number of each z-stack layer is shown on the top of the image. DAPI, Nucleolin and AGO1x signals correspond to blue, red and green components of images, respectively. Detected outlines of the nucleus and nucleoli are shown in magenta and white color, respectively. **C,** Histogram of AGO1x intensity in the areas labeled as nucleoplasm and nucleoli in panel (B).

Appendix Fig S4 Characterization of AGO1x depleted mutants by sequencing.

A-B, DNA sequencing traces for the two mutant cell lines in MDA-MB-231 (A) and HeLa (B). A reverse sequencing primer (i.e. 3’→5’) targeting the 3’ end of region encoding AGO1x was used to confirm DNA editing in the CRISPR/Cas9 mutants. Traces for W1A (middle) and W6A (bottom) have been aligned to the reference unedited genomic region (top). Bases highlighted in red or yellow do not match the reference sequence. Different colors in the chromatogram peaks represent different DNA sequence identities. Amino acids encoded by the translation readthrough region are also depicted in the grey boxes. **C-D,** Alignment of RNA-seq reads from control and mutant MDA-MB-231 (C) and HeLa (D) cell lines to the 3’ UTR region of AGO1 that is subject to translational readthrough and was targeted by the sgRNAs. Deletions observed in the RNA-seq from mutant lines are indicated by dashes. **E,** Representative western blots demonstrating the depletion of AGO1x in three replicates of mutant compared to control MDA-MB-231 cell lines. This is shown both by the reduction in intensity of the higher MW

observed in the RNA-seq from mutant lines are indicated by dashes. **E,** Representative western blots demonstrating the depletion of AGO1x in three replicates of mutant compared to control MDA-MB-231 cell lines. This is shown both by the reduction in intensity of the higher MW band (marked with an arrow) when the gel was probed with AGO1 antibody, as well as by the reduction in intensity of the band specifically recognized by the AGO1x antibody used to reprobe the gel. The levels of canonical AGO1 were not significantly altered. **F,** Quantification of AGO1 (upper panel) and AGO1x (lower panels) levels in control and edited cells, normalized to the GAPDH loading control. For comparison of multiple blots, the values obtained for control lines were taken as unit reference. P-values were calculated from unpaired two-tailed *t*-tests.

Appendix Fig S5 AGO1x deletion impairs the growth of HeLa cells.
A, Western blot analysis of mutant W1A, W6A and control HeLa cell lines, using AGO1 and AGO1x antibodies. GAPDH served as an endogenous control for loading. **B**, Representative phase contrast images illustrate the growth patterns of control and mutant HeLa cell lines at 24 hr and 72 hr after seeding equal numbers of each cell type in wells of a six-well plate. **C**, Impedance-based mean (+/- s.d.) cell indices at the indicated time points after seeding equal numbers of control (n=6), W1A (n=5) and W6A (n=5) HeLa cells. From 24 hours on, there is a statistical significant difference between control and the two mutants ($P < 0.005$, two tailed *t*-test).

Appendix Fig S6 Deletion of AGO1x in HeLa cells activates the interferon response.
A-B, Scatter plots of mean log10 mRNA expression levels (transcript counts per million, TPM) in W1A (A) and W6A mutant cell lines (B) compared to control (n=3). mRNAs that are significantly upregulated or downregulated (|fold-change| > 2-fold and FDR < 0.01) in the mutant cell lines are shown in red and blue, respectively.

Appendix Fig S7 Loss of AGO1x expression leads to Caspase activation and PARP cleavage.
A, Scheme representing the use of fluorescent substrates to measure the activity of Caspases 8/9/3 in a multiplexed, single-well reaction. **B-C**, Dot plot representing the average signal from individual caspases 12 hours after seeding control and mutant MDA-MB-231 (B) and HeLa (C) cells in 96 well plate format. At least 10 biological replicates of the cell types were used for comparison. *P*-values represent results of two-tailed *t*-test performed on each mutant cell type relative to its corresponding control cell type. **D**, Western blot analysis of apoptosis markers in cellular lysates of control and AGO1x-depleted mutant cell lines. The same lysates were used in parallel blots (each blot indicated by a vertical line) to assess the levels of PARP, p53 and NFKB (p65/RELA), as well as of the total and phosphorylated (Ser-51) translation initiation factor eIF2α. GAPDH levels serve as loading control for each blot. The image of the GAPDH loading control for the eIF2α blot is identical to that in the blot of Fig. 3A, as both eIF2α, AGO1x were probed on the same membrane. The lower panel shows quantifications of total eIF2α and phospho-eIF2α (Ser-51) levels. The intensity of each band relative to the corresponding GAPDH control was first calculated and then values were normalized to the

Appendix Fig S8 PNPT1 partially localizes to the nucleus and interacts with AGO1x independently of RNA

A, Representative IF images of PNPT1 expression (green), showing the close proximity and partial overlap with the nucleus (DAPI, red). **B,** 2.5D reconstruction of a particular field of view of PNPT1 and DAPI-stained cells to demonstrate the overlap of signals (in yellow, from Zeiss ZEN software). **C**, Analysis of PNPT1 immunoprecipitate from MDA-MB-231 cells. Cell lysates were treated with either RNase A or mock at 37 degrees for one hour prior to IP. Western blot analysis of the eluted proteins after IP confirms that PNPT1-AGO1x interaction is partly stable with respect to the treatment with RNase A, which cleaves ssRNA and dsRNA.

Appendix Fig S9 Protein interactome analysis of FLAG-tagged AGO1 and AGO1x proteins from whole cell lysates.

A, Scatter plot of mean spectral counts for proteins that co-immunoprecipitated with FLAG-AGO1/AGO1x from two cell lines (MDA-MB-231 and HeLa) that expressed ectopically FLAG-tagged constructs of AGO1 or AGO1x (stop codon modified to serine-encoding codon) (n=4). Targets indicated in blue are significantly depleted in the AGO1x IP (FDR < 0.01) . The group of TNRC6 (GW182) proteins show a marked depletion in three biological replicates of FLAG-AGO1x-IP compared to FLAG-AGO1-IP. **B,** The difference in interaction in MDA-MB-231 cells was verified with a commercial antibody for TNRC6B obtained from Bethyl Laboratories. All IP experiments were performed with FLAG beads (M8823-1ML) available from Sigma and experimental steps were processed according to the manufacturer's protocol.

Appendix Fig S10 RNA binding propensity of AGO1

A, Schema of fractionation of MDA-MB-231 control cells and IP with AGO1 antibody from the cytosolic and nuclear fractions. **B,** Absolute C_t values for putative AGO1x targets and non-targets, amplified from two biological replicates of AGO1-IP from cytosol and nuclear fraction of MDA-MB-231 control cells. Instances when only one data point is plotted indicate that no amplification was obtained from the other biological replicate of the same fraction (RNA not detected).

Appendix Fig S11 Effect of AGO1x on cellular ribosomes and protein translation

A-B, qRT-PCR-based estimation of 45S pre-rRNA and mature ribosomal RNA levels in control and AGO1x mutant cell lines. Barplots represent mean (+/- s.d.) relative levels normalized to GAPDH expression from three independent biological replicates in MDA-MB-231 (A) and two biological replicates in HeLa (B). Shown are also *P*-values given by the unpaired two-tailed Student's *t*-test comparing mutant and the control lines. **C,** Representative overlayed profiles of 40S and 60S ribosomal subunits in MDA-MB-231 control (blue line), W1A (black line) and W6A (red line) samples, after dissociating the ribosomes by adding 40 mM EDTA in both polysome lysis buffer and gradient solution. **D,** Summary of relative 40S/60S ratio in W1A and W6A samples in comparison to MDA-MB-231 control samples. Dots represent the normalized ratio of the area under the curve (AUC) for 40S and 60S subunit peaks from four biological replicates of each cell line. Lines indicate the mean and +/- one s.d. P-values for two-tailed *t*-tests comparing the ratio between the indicated samples are shown. The AUC was calculated using the ImageJ 1.52a software (NIH). **E,** Distribution of label

intensity in HeLa cells under excitation with the 561 laser, BP filter 610 /20 laser, following the incorporation of homopropargylglycine (HPG) and click labelling, to measure the overall protein synthesis rate. X-axis indicates the signal intensity of incorporated HPG, Y-axis the relative frequency of cells with corresponding signal intensity. Two independent biological replicates of control and W1A and W6A cells were analyzed in a BD LSR Fortessa FACS instrument. The number shown in each panel is the median signal intensity. The histograms were generated with the FlowJo software (BD Biosciences). For reference, the median signal intensity of unstained HeLa cells was 9.49 (not shown). **F,** Distribution of label intensity in MDA-MB-231 cells under excitation with the 561 laser, BP filter 610 /20 laser, following the incorporation of homopropargylglycine (HPG) and click labelling. Three independent biological replicates were analyzed in a BD LSR Fortessa FACS instrument. X and Y axes as in panel E. The number shown in each panel is the median signal intensity, which for unstained cells was 32.5 (not shown).

A

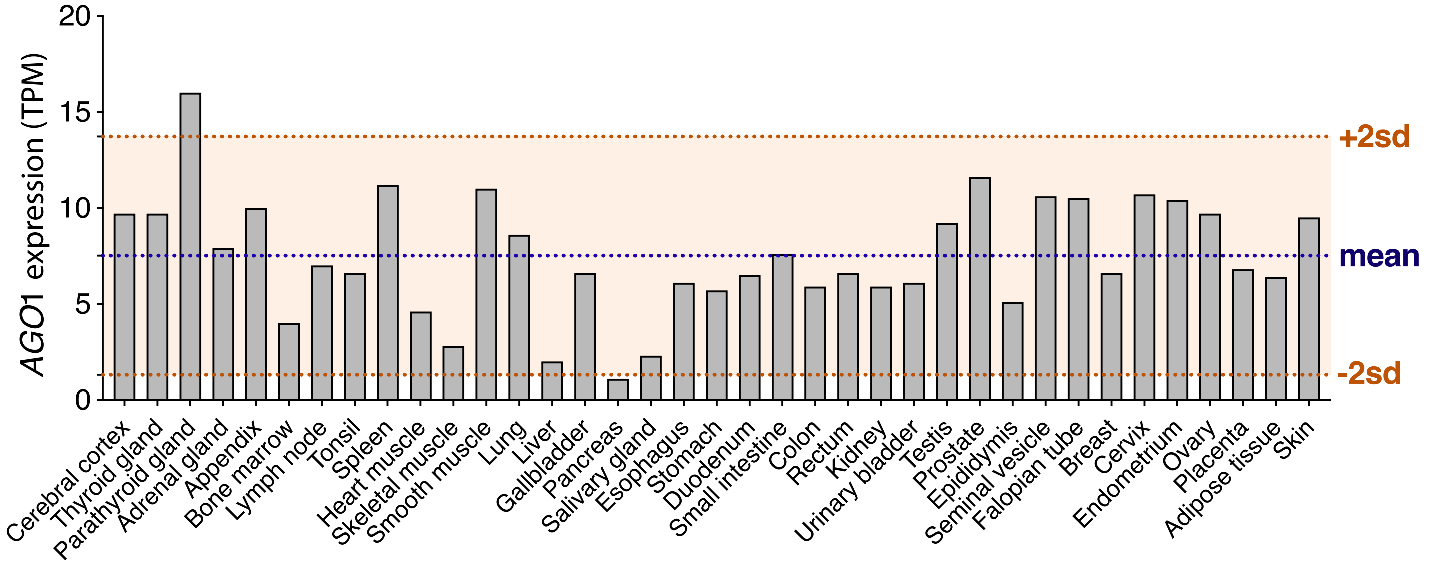

20
15
10
5
0
AGO1 expression (TPM)
+2sd
mean
-2sd
Cerebral cortex
Thyroid gland
Parathyroid gland
Adrenal gland
Appendix
Bone marrow
Lymph node
Tonsil
Spleen
Heart muscle
Skeletal muscle
Smooth muscle
Lung
Liver
Gallbladder
Pancreas
Salivary gland
Esophagus
Stomach
Duodenum
Small intestine
Colon
Rectum
Kidney
Urinary bladder
Testis
Prostate
Epididymis
Seminal vesicle
Falopian tube
Breast
Cervix
Endometrium
Ovary
Placenta
Adipose tissue
Skin

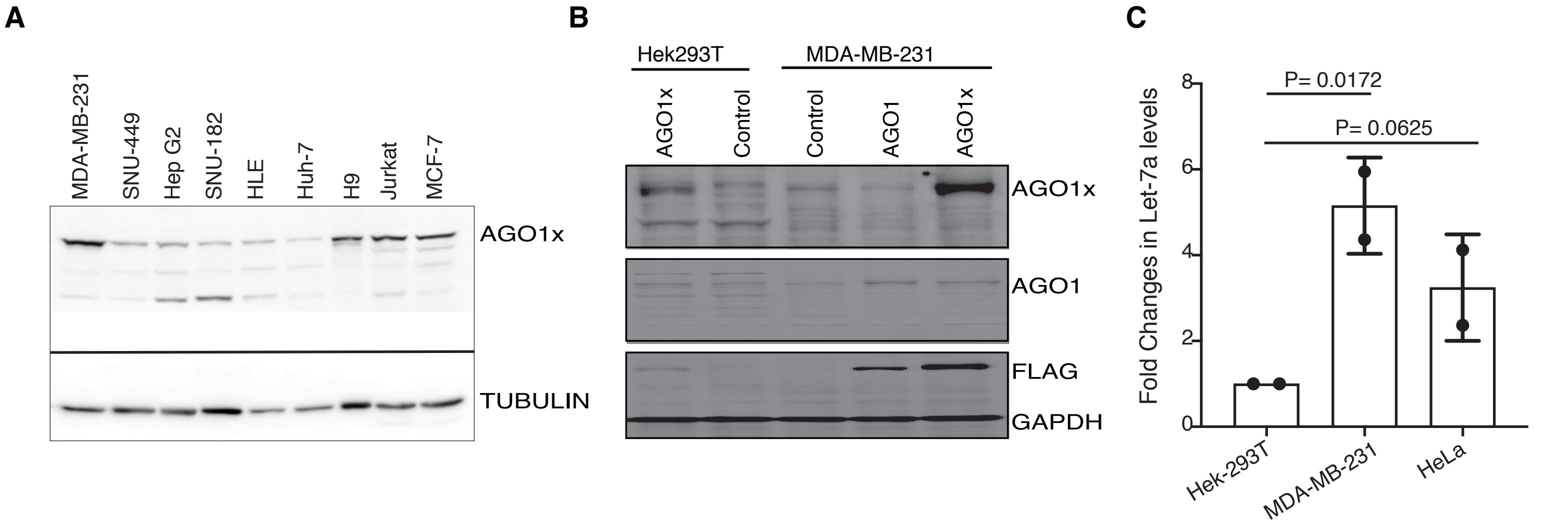
A
MDA-MB-231
SNU-449
Hep G2
SNU-182
HLE
Huh-7
H9
Jurkat
MCF-7
AGO1x
TUBULIN
B
Hek293T
MDA-MB-231
AGO1x
Control
Control
AGO1
AGO1x
AGO1x
AGO1
FLAG
GAPDH
C
P= 0.0172
P= 0.0625
Fold Changes in Let-7a levels
8
6
4
2
0
Hek-293T
MDA-MB-231
HeLa

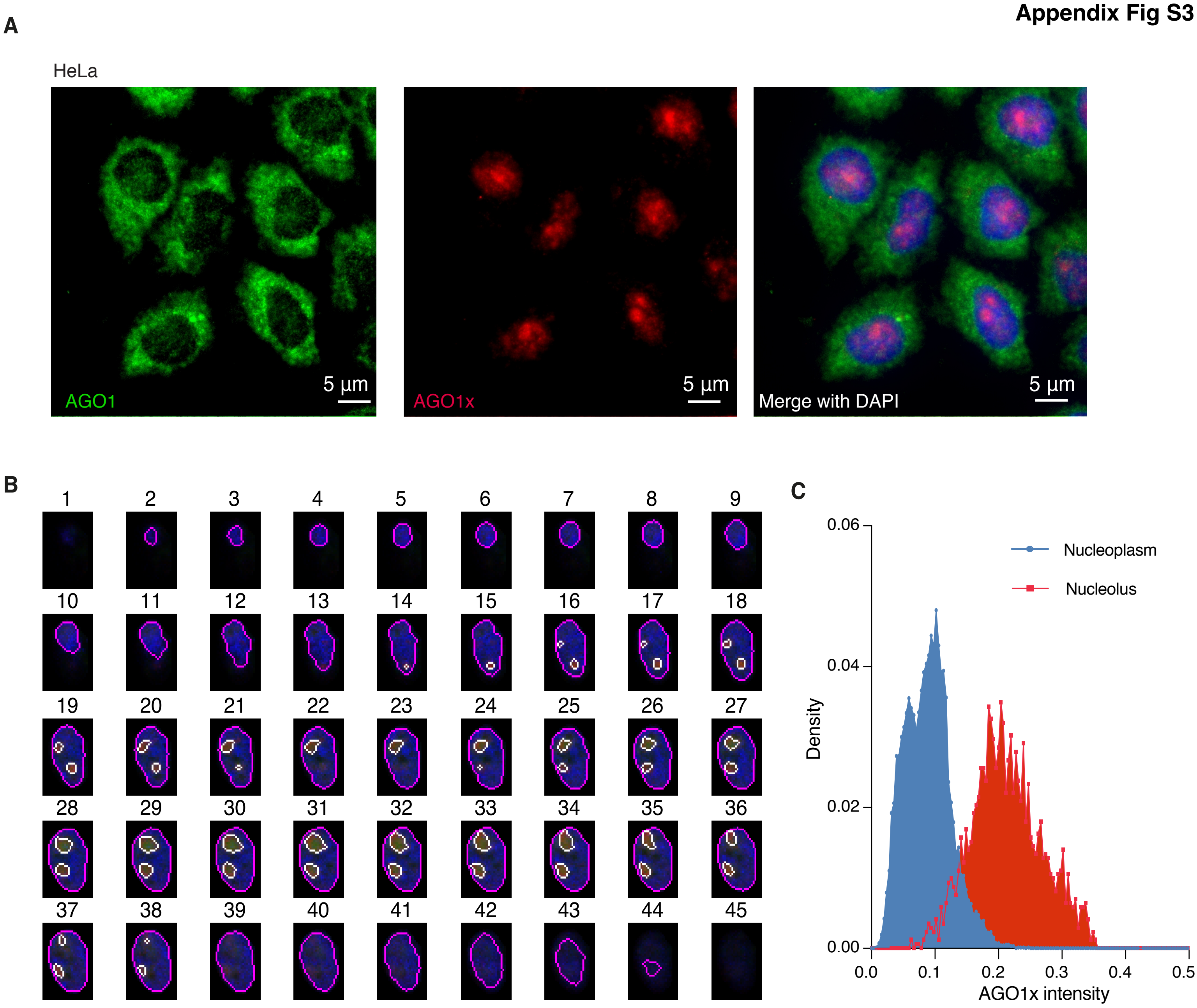
A
HeLa
AGO1
5 µm
AGO1x
5 µm
Merge with DAPI
5 µm
B
1 2 3 4 5 6 7 8 9
10 11 12 13 14 15 16 17 18
19 20 21 22 23 24 25 26 27
28 29 30 31 32 33 34 35 36
37 38 39 40 41 42 43 44 45
C
Nucleoplasm
Nucleolus
Density
0.00
0.02
0.04
0.06
0.0 0.1 0.2 0.3 0.4 0.5
AGO1x intensity

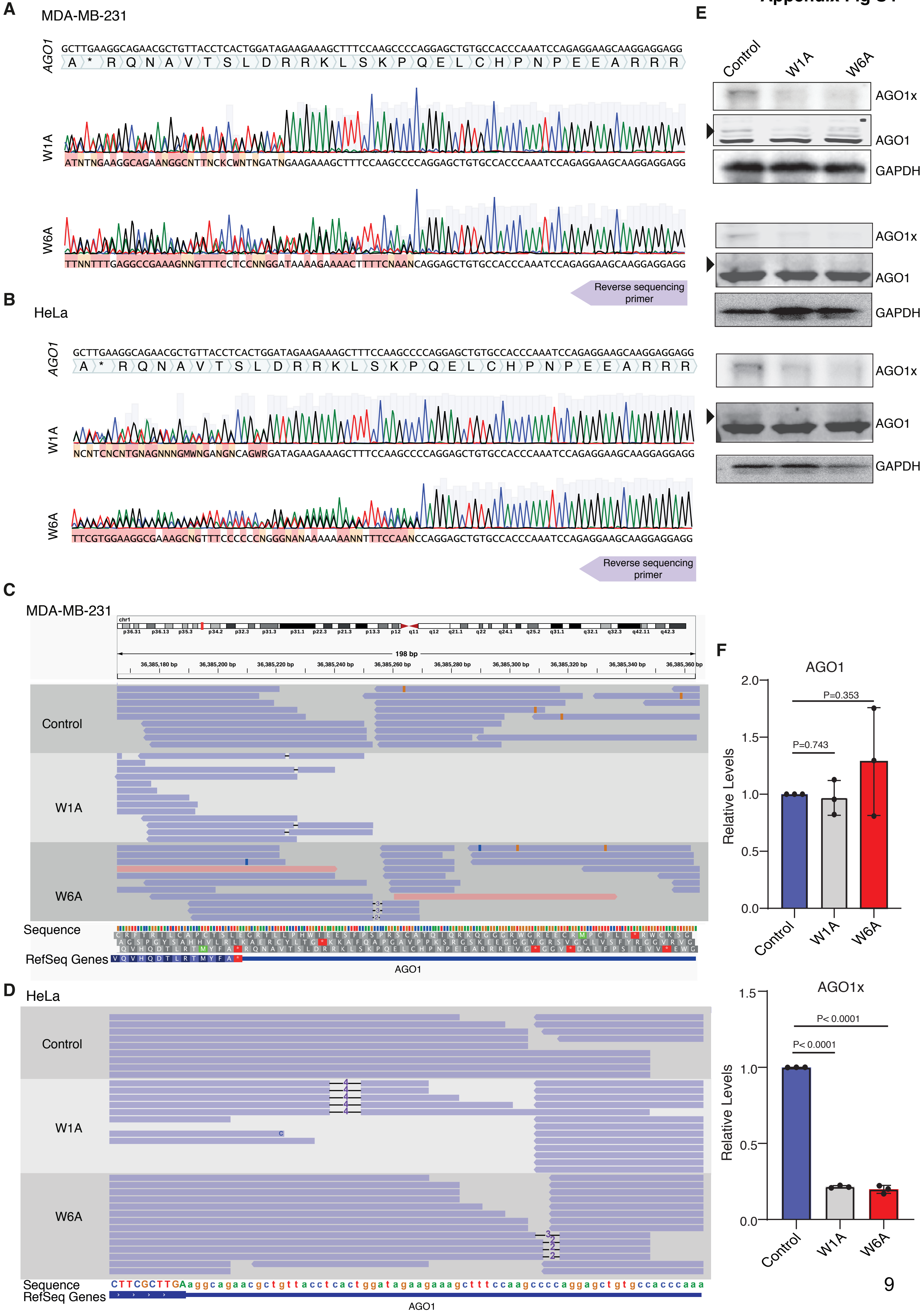
A
MDA-MB-231
AGO1
GCTTGAAGGCAGAACGCTGTTACCTCACTGGATAGAAGAAAGCTTTCCAAGCCCCAGGAGCTGTGCCACCCAAATCCAGAGGAAGCAAGGAGGAGG
W1A
W6A
Reverse sequencing primer
B
HeLa
AGO1
W1A
W6A
Reverse sequencing primer
C
MDA-MB-231
198 bp
Control
W1A
W6A
Sequence
RefSeq Genes
AGO1
D
HeLa
Control
W1A
W6A
Sequence
RefSeq Genes
AGO1
E
Control
W1A
W6A
AGO1x
AGO1
GAPDH
F
AGO1
P=0.353
P=0.743
Relative Levels
Control
W1A
W6A
AGO1x
P< 0.0001
P< 0.0001
Relative Levels
Control
W1A
W6A

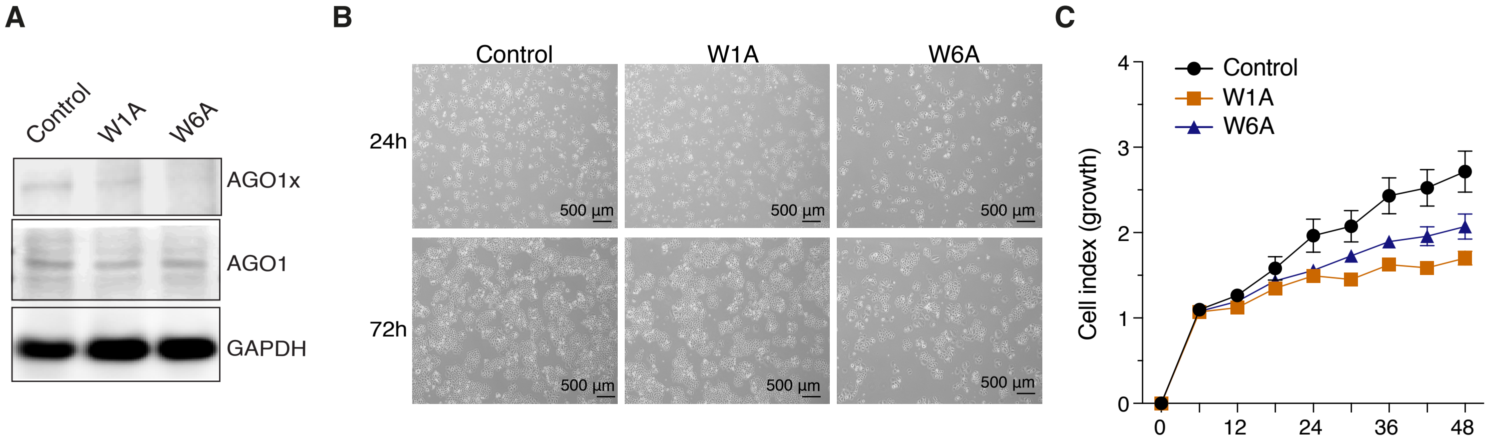
A
Control
W1A
W6A
AGO1x
AGO1
GAPDH
B
Control
W1A
W6A
24h
72h
500 µm
C
Control
W1A
W6A
Cell index (growth)
0
1
2
3
4
0
12
24
36
48

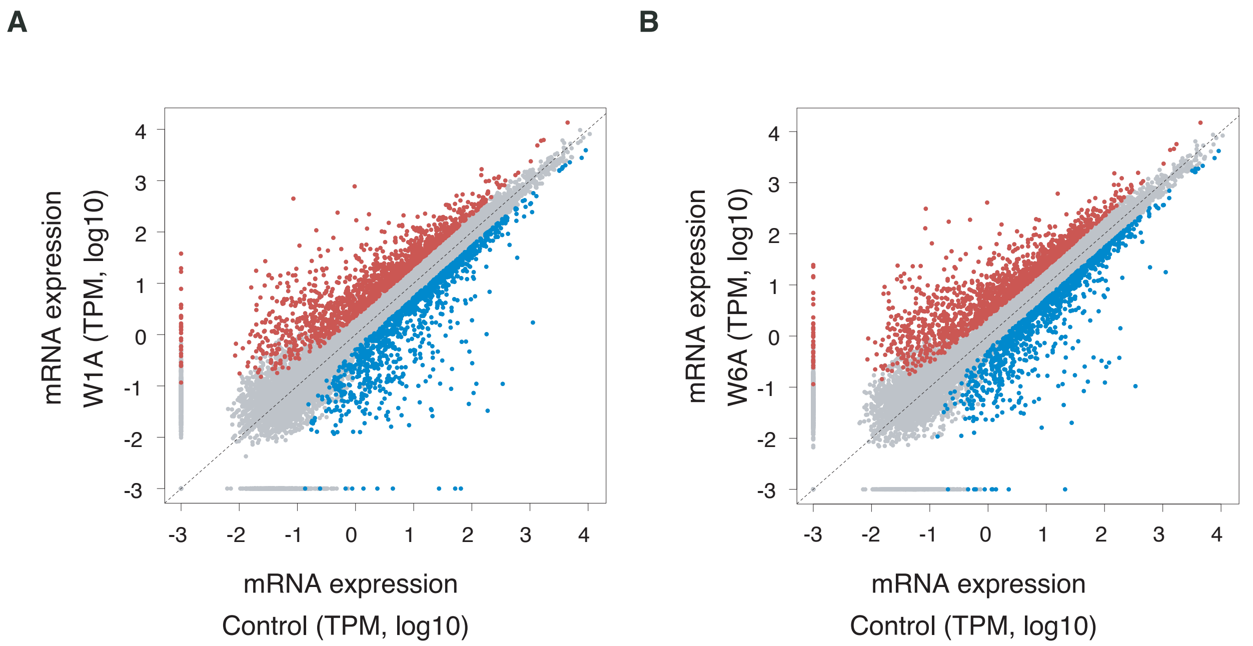
A
B
mRNA expression
W1A (TPM, log10)
mRNA expression
W6A (TPM, log10)
4
3
2
1
0
-1
-2
-3
-3
-2
-1
0
1
2
3
4
mRNA expression
Control (TPM, log10)
mRNA expression
Control (TPM, log10)

Appendix Fig S7

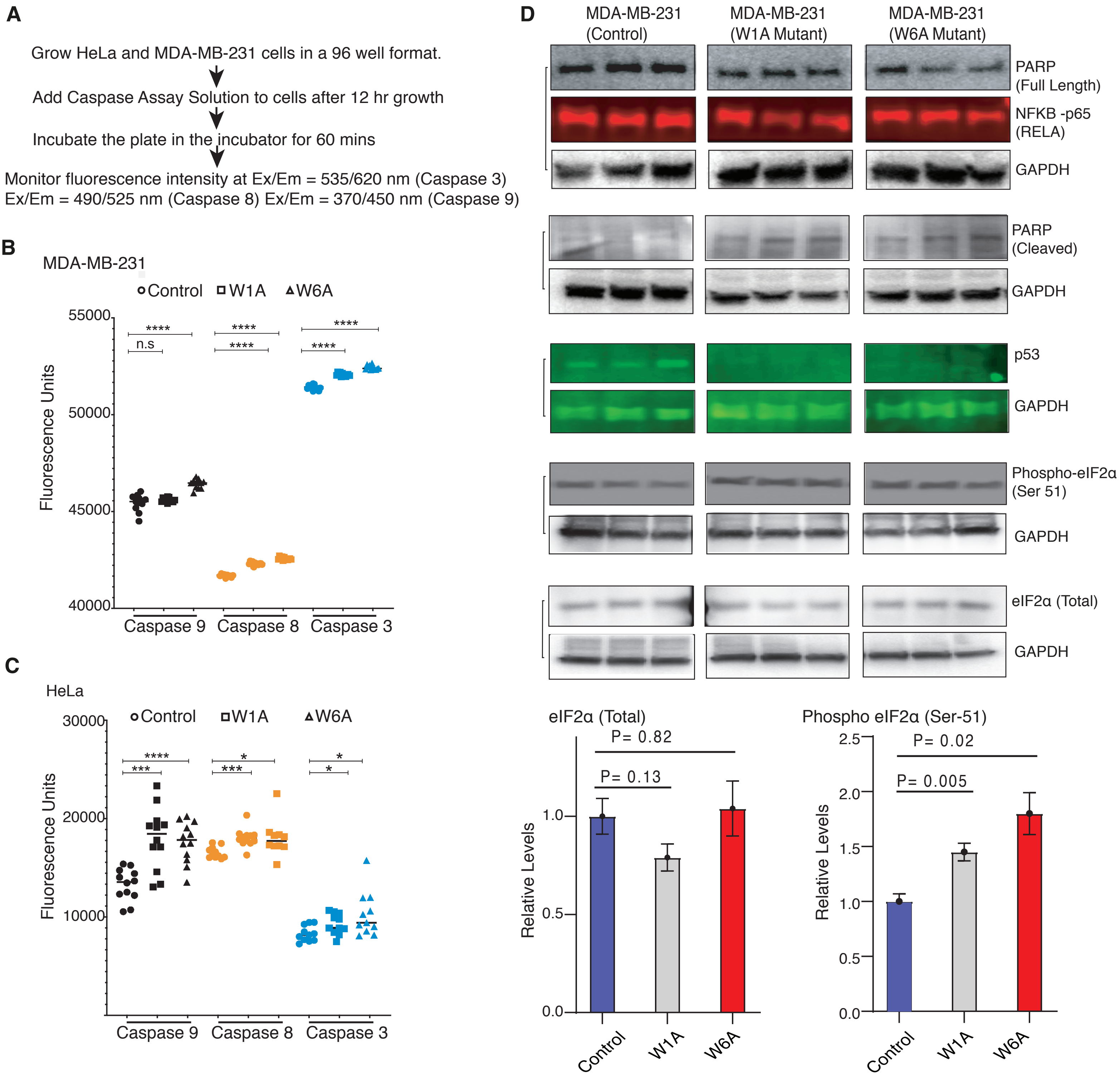

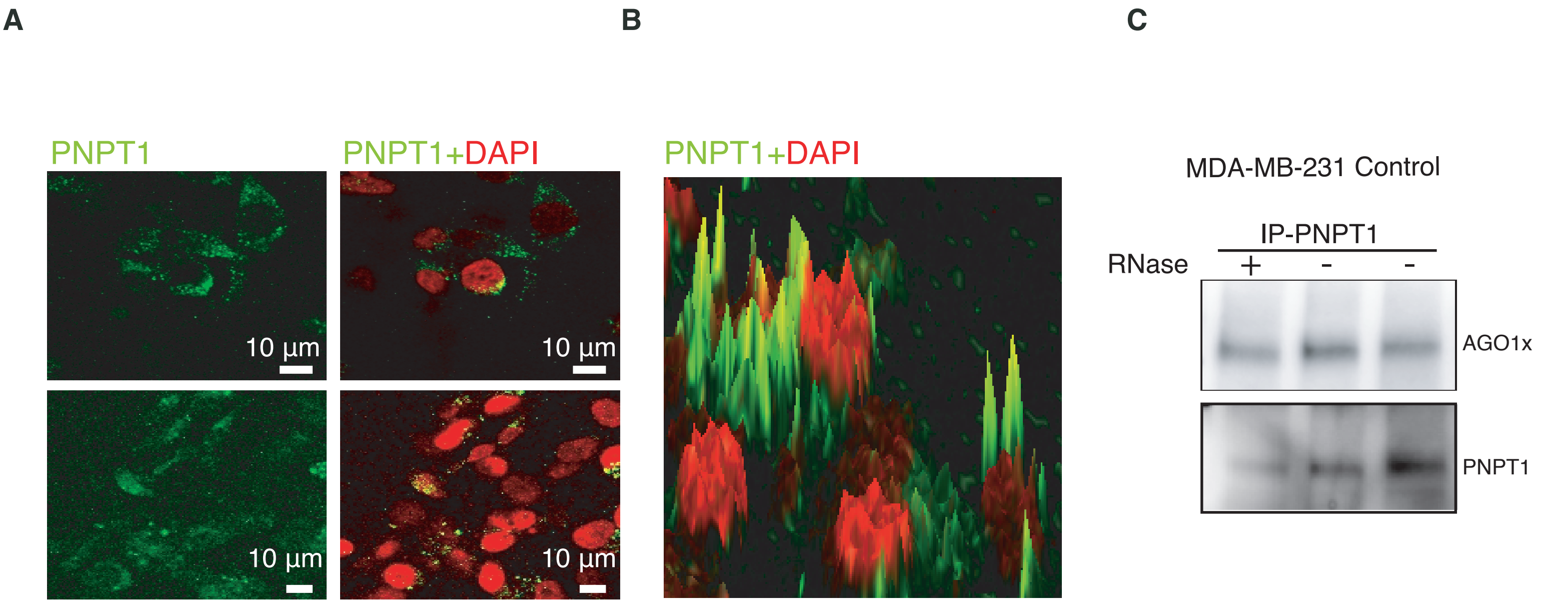
A
B
C
PNPT1
PNPT1+DAPI
10 µm
10 µm
10 µm
10 µm
PNPT1+DAPI
MDA-MB-231 Control
IP-PNPT1
RNase
+
-
-
AGO1x
PNPT1

A

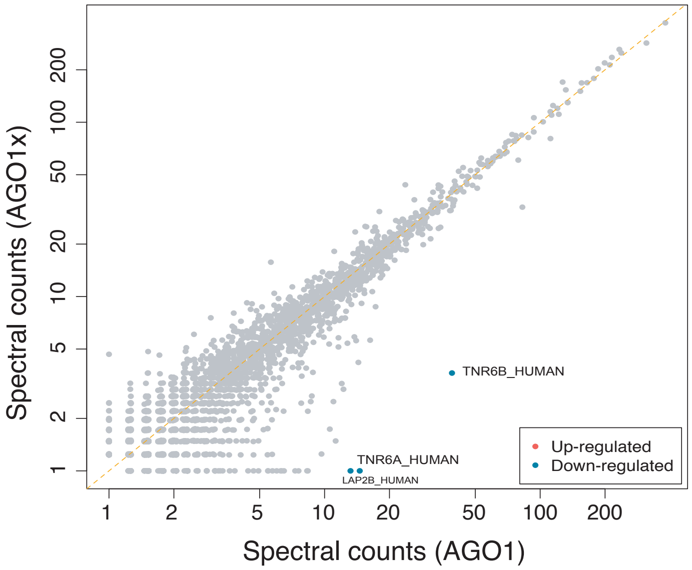
Spectral counts (AGO1x)
Spectral counts (AGO1)
1
2
5
10
20
50
100
200
TNR6B_HUMAN
TNR6A_HUMAN
LAP2B_HUMAN
Up-regulated
Down-regulated

B

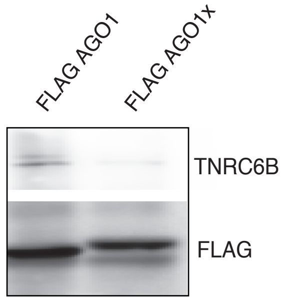
FLAG AGO1
FLAG AGO1x
TNRC6B
FLAG

A

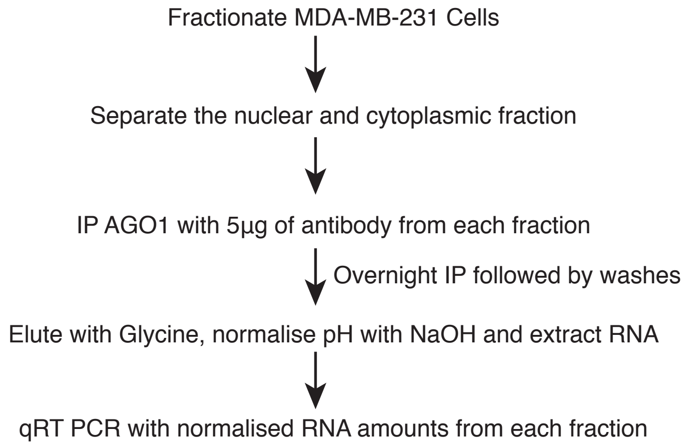
Fractionate MDA-MB-231 Cells
Separate the nuclear and cytoplasmic fraction
IP AGO1 with 5µg of antibody from each fraction
Overnight IP followed by washes
Elute with Glycine, normalise pH with NaOH and extract RNA
qRT PCR with normalised RNA amounts from each fraction

B

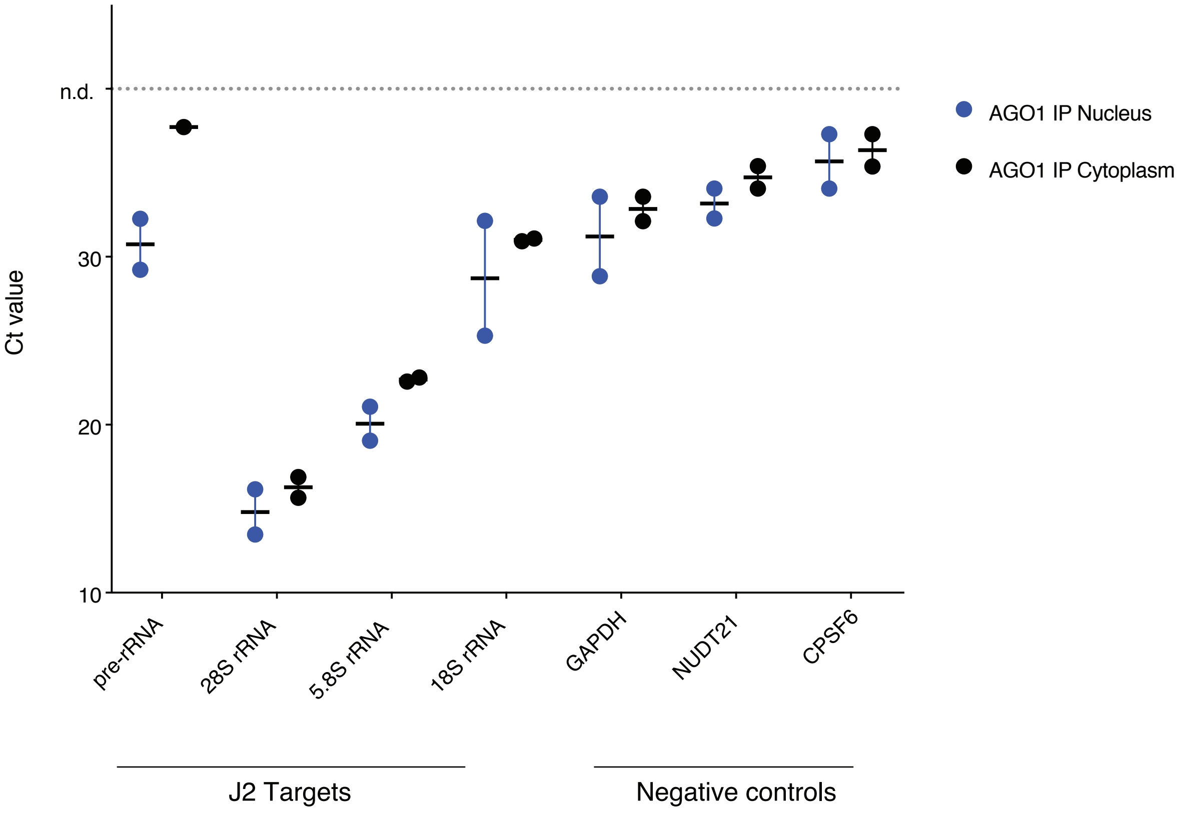
Ct value
n.d.
30
20
10
pre-rRNA
28S rRNA
5.8S rRNA
18S rRNA
GAPDH
NUDT21
CPSF6
J2 Targets
Negative controls
AGO1 IP Nucleus
AGO1 IP Cytoplasm

Appendix Fig S11

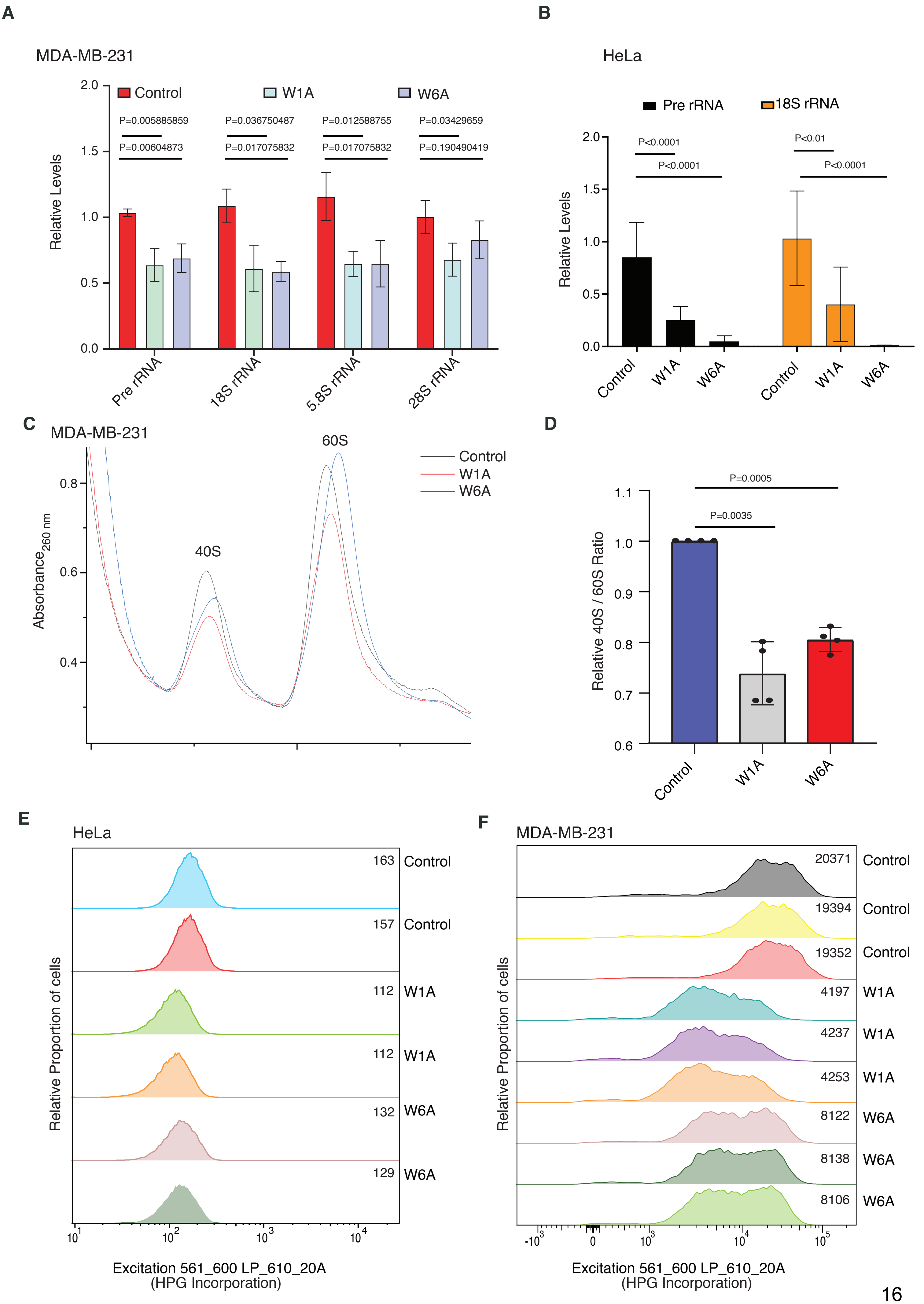

Appendix References

Uhlén M, Fagerberg L, Hallström BM, Lindskog C, Oksvold P, Mardinoglu A, Sivertsson Å, Kampf C, Sjöstedt E, Asplund A, Olsson I, Edlund K, Lundberg E, Navani S, Szigyarto CA-K, Odeberg J, Djureinovic D, Takanen JO, Hober S, Alm T, et al (2015) Proteomics. Tissue-based map of the human proteome. *Science* **347:** 1260419
