# Supplementary material for: Prevention of dsRNA‐induced interferon signaling by AGO1x is linked to breast cancer cell proliferation
Source: EMBO J. 2020 Aug 19;39(18):e103922. doi: 10.15252/embj.2019103922 (PMC7507497; doi:10.15252/embj.2019103922)
Supplement: Supplementary file 2 — Expanded View Figures PDF [file EMBJ-39-e103922-s002.pdf]

Expanded View Figures

◀ **Figure EV1. AGO1x antibody targets specifically its cognate protein and not the canonical AGO1.**

A Western blot analysis of multiple cell lines demonstrates that in addition to the canonical AGO1 protein band, a characteristic second band of higher MW is revealed by the AGO1 antibody.

B Higher MW band observed in (A) corresponds to AGO1x protein. Representative Western blot shows that the intensity of the higher MW band is sensitive to ectopic overexpression (using the pIRES-Neo vector) of FLAG-tagged AGO1x but not of FLAG-tagged AGO1. Expression of the corresponding isoform is confirmed with a blot for FLAG expression. The overexpression constructs are indicated with labels above the blots. Protein ladders show that the higher MW band corresponds to approximately 100 kDa.

C, D Western blot with AGO1x antibody demonstrates its specificity for the AGO1x isoform stably expressed from pCDH-FLAG-tagged plasmids. Middle and lower panels depict AGO1x levels in individual samples, at low and high exposure, respectively, of the same blot. The higher exposure was used to better assess the expression level in untransfected (control) samples.

E, F Representative images of MDA-MB-231 stained with AGO1 (green) and AGO1x (red) antibodies under conditions of endogenous expression or knockdown with an siRNA pool targeting the transcript that encodes both isoforms (E). An AGO1x overexpression system, where FLAG-AGO1x was stably integrated into MDA-MB-231 cells, was also tested (F). DAPI was used to mark the nucleus (blue).

G Western blot analysis of AGO1x protein level in cells treated with either siAGO1 or siControl confirms the imaging results from panel E.

Source data are available online for this figure.

 © 2020 The Authors

24 of 23 The EMBO Journal e103922 | 2020 © 2020 The Authors

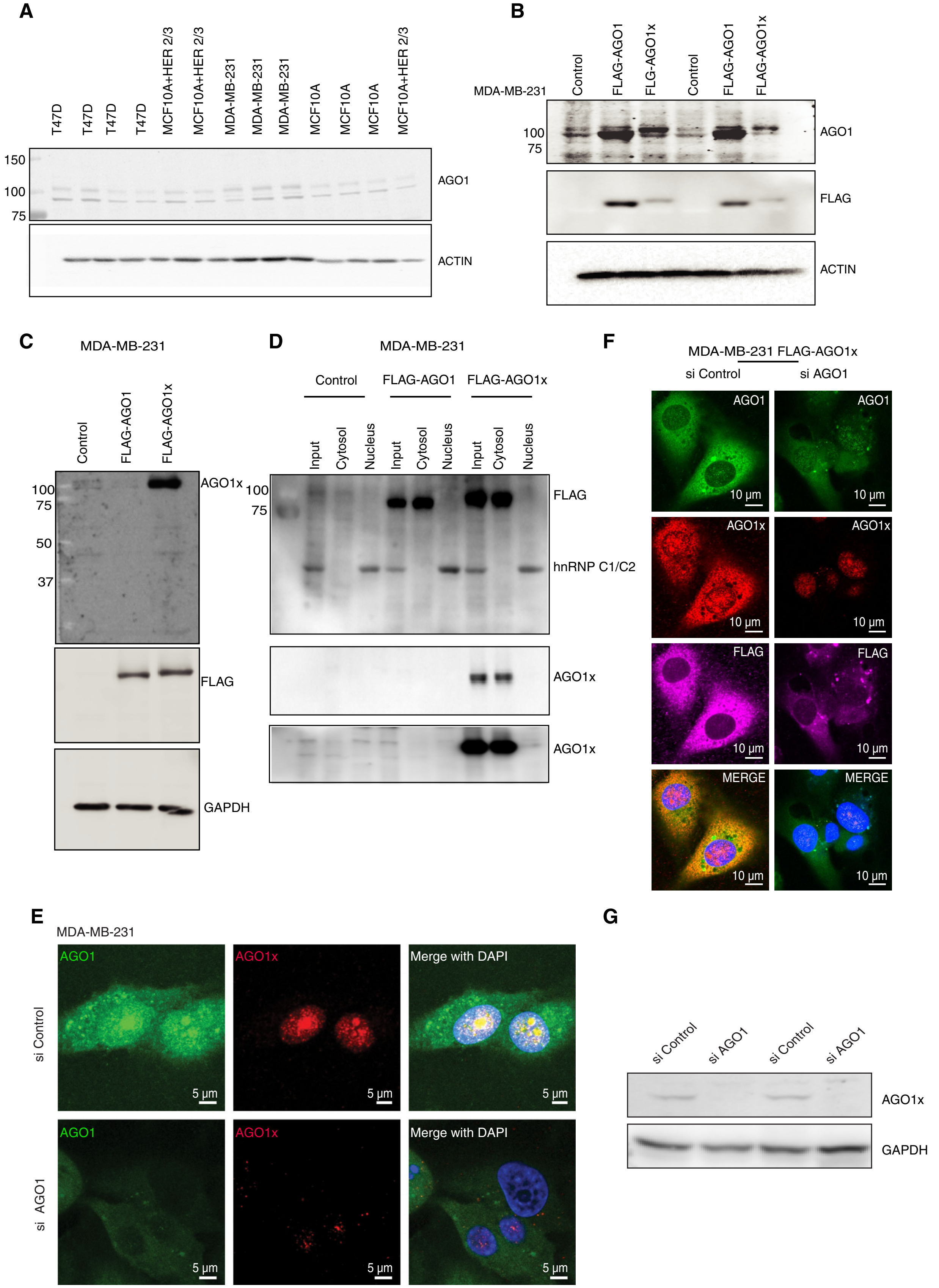
A
T47D
T47D
T47D
T47D
MCF10A+HER 2/3
MCF10A+HER 2/3
MDA-MB-231
MDA-MB-231
MDA-MB-231
MCF10A
MCF10A
MCF10A
MCF10A+HER 2/3
150
100
75
AGO1
ACTIN
B
MDA-MB-231
Control
FLAG-AGO1
FLG-AGO1x
Control
FLAG-AGO1
FLAG-AGO1x
100
75
AGO1
FLAG
ACTIN
C
MDA-MB-231
Control
FLAG-AGO1
FLAG-AGO1x
100
75
50
37
AGO1x
FLAG
GAPDH
D
MDA-MB-231
Control
FLAG-AGO1
FLAG-AGO1x
Input
Cytosol
Nucleus
100
75
FLAG
hnRNP C1/C2
AGO1x
AGO1x
F
MDA-MB-231 FLAG-AGO1x
si Control
si AGO1
AGO1
AGO1x
FLAG
MERGE
10 µm
E
MDA-MB-231
si Control
si AGO1
AGO1
AGO1x
Merge with DAPI
5 µm
G
si Control
si AGO1
si Control
si AGO1
AGO1x
GAPDH

Figure EV1.

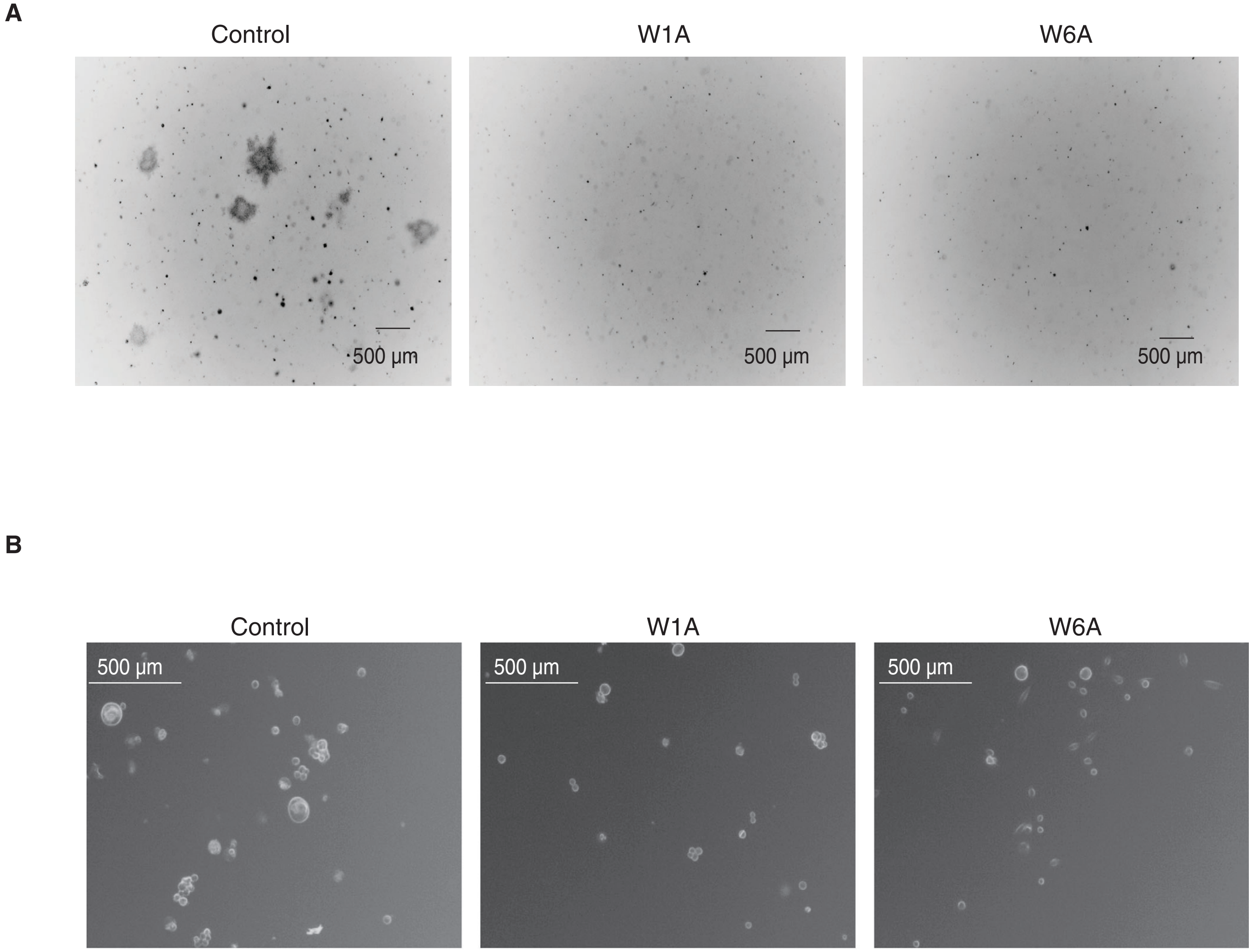

Figure EV2. Phenotypical consequences of AGO1x depletion in MDA-MB-231 cells.

A Representative images of colony formation assays performed with the W1A, W6A, and control MDA-MB-231 cell lines. Images were captured with an inverted microscope (ZEISS Axio Vert. A1 equipped with an AxioCam MRc camera).

B Representative images of sphere formation assays performed with W1A, W6A, and control MDA-MB-231 cell lines. Images were captured with an inverted phase-contrast microscope (Leica) and were inverted for improved contrast.

 © 2020 The Authors

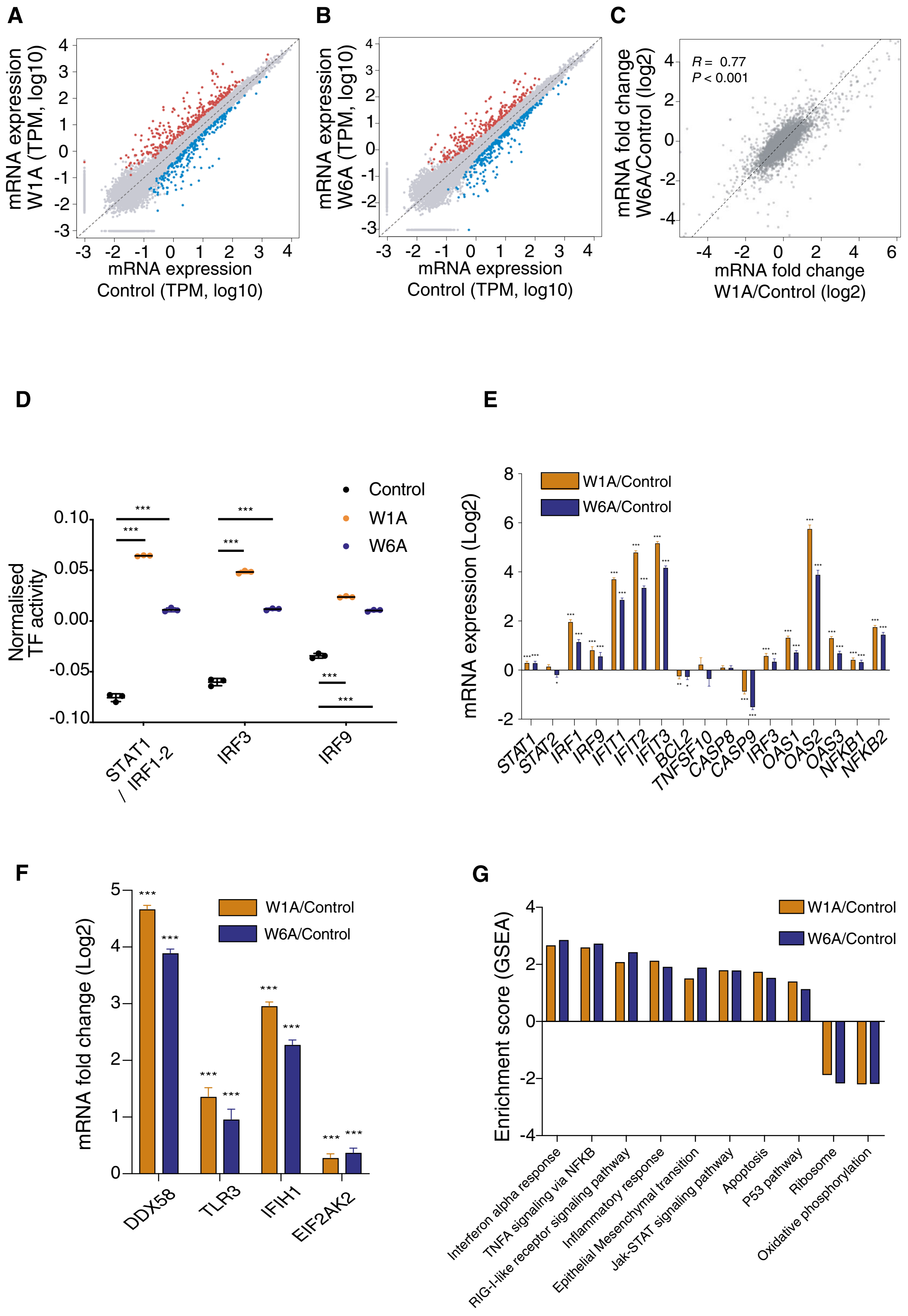
A
mRNA expression W1A (TPM, log10)
mRNA expression Control (TPM, log10)
B
mRNA expression W6A (TPM, log10)
mRNA expression Control (TPM, log10)
C
R = 0.77
P < 0.001
mRNA fold change W6A/Control (log2)
mRNA fold change W1A/Control (log2)
D
Control
W1A
W6A
Normalised TF activity
STAT1 / IRF1-2
IRF3
IRF9
E
W1A/Control
W6A/Control
mRNA expression (Log2)
STAT1 STAT2 IRF1 IRF9 IFIT1 IFIT2 IFIT3 BCL2 TNFSF10 CASP8 CASP9 IRF3 OAS1 OAS2 OAS3 NFKB1 NFKB2
F
W1A/Control
W6A/Control
mRNA fold change (Log2)
DDX58
TLR3
IFIH1
EIF2AK2
G
W1A/Control
W6A/Control
Enrichment score (GSEA)
Interferon alpha response
TNFA signaling via NFKB
RIG-I-like receptor signaling pathway
Inflammatory response
Epithelial Mesenchymal transition
Jak-STAT signaling pathway
Apoptosis
P53 pathway
Ribosome
Oxidative phosphorylation

Figure EV3.

Figure EV3. Deletion of AGO1x in HeLa cells activates the interferon response.

A, B Scatter plots of mean log10 mRNA expression levels (in transcript counts per million, TPM) in W1A (A) and W6A mutant cell lines (B) compared to the control line ($n = 3$). mRNAs that are significantly upregulated or downregulated (|fold change| > 2 and FDR < 0.01) in the mutant cell lines are shown in red and blue, respectively. The dashed line indicates equal mRNA levels for control and mutant cell lines.

C Scatter plot of mean log2 mRNA fold changes from three biological replicates in the two mutant cell lines compared to control. Shown is also the Pearson correlation coefficient and respective *P*-value. Dashed line indicates equal change in the two mutant lines.

D Mean (± SD) activity of STAT1/IRF1/IRF2/IRF3/IRF9 transcription factor motifs estimated by ISMARA (Balwierz *et al*, 2014) from the RNA-seq data ($n = 3$). Shown are the *P*-values (***$P < 0.001$) determined by Dunn's multiple comparison test post hoc and the non-parametric Kruskal–Wallis test ($P < 0.0001$).

E, F Mean (± SEM) log2 mRNA expression fold changes of genes involved in the interferon-alpha response and apoptosis (E) and in dsRNA sensing (F) in the two mutant cell lines relative to control ($n = 3$). Multiple testing-corrected *P*-values for fold changes with respect to control are depicted above each bar (*$P < 0.05$, **$P < 0.01$, ***$P < 0.001$).

G Normalized enrichment score (ES) from gene set enrichment analysis comparing gene expression in the two mutant cell lines with that in the control cell line. For all the pathways depicted, $P < 0.05$. *P*-values were calculated by comparing the empirical ES of a gene set relative to a null distribution of ESs derived from permuting the gene set and then adjusted for multiple hypothesis testing.

Source data are available online for this figure.

Figure EV4. AGO1x deficiency triggers reduction of SINV accumulation in Hela cells.

A GFP fluorescent microscopy images of control and mutant cell lines infected at MOI of 0.1 or 1 with SINV-GFP virus for 48 h. The left panel corresponds to the GFP signal from infected cells and the right panel to a merge of GFP signal and bright field. Pictures were taken with a 5× magnification. MOI: multiplicity of infection; hpi: hours post-infection.

B Representative Western blot of p-PKR, p-eIF2alpha (Ser-52), and GFP expression in SINV-GFP-infected cells in the same condition as in (A). Tubulin was used as loading control.

C Mean (± SEM) of SINV-GFP viral titers in control and mutant lines infected at an MOI of 0.1 or 1 for 48 h ($n = 3$) from plaque assay quantification. **$P < 0.001$. *P*-value was obtained using an ordinary one-way ANOVA test comparing mutant cell lines to control.

Source data are available online for this figure.

 © 2020 The Authors

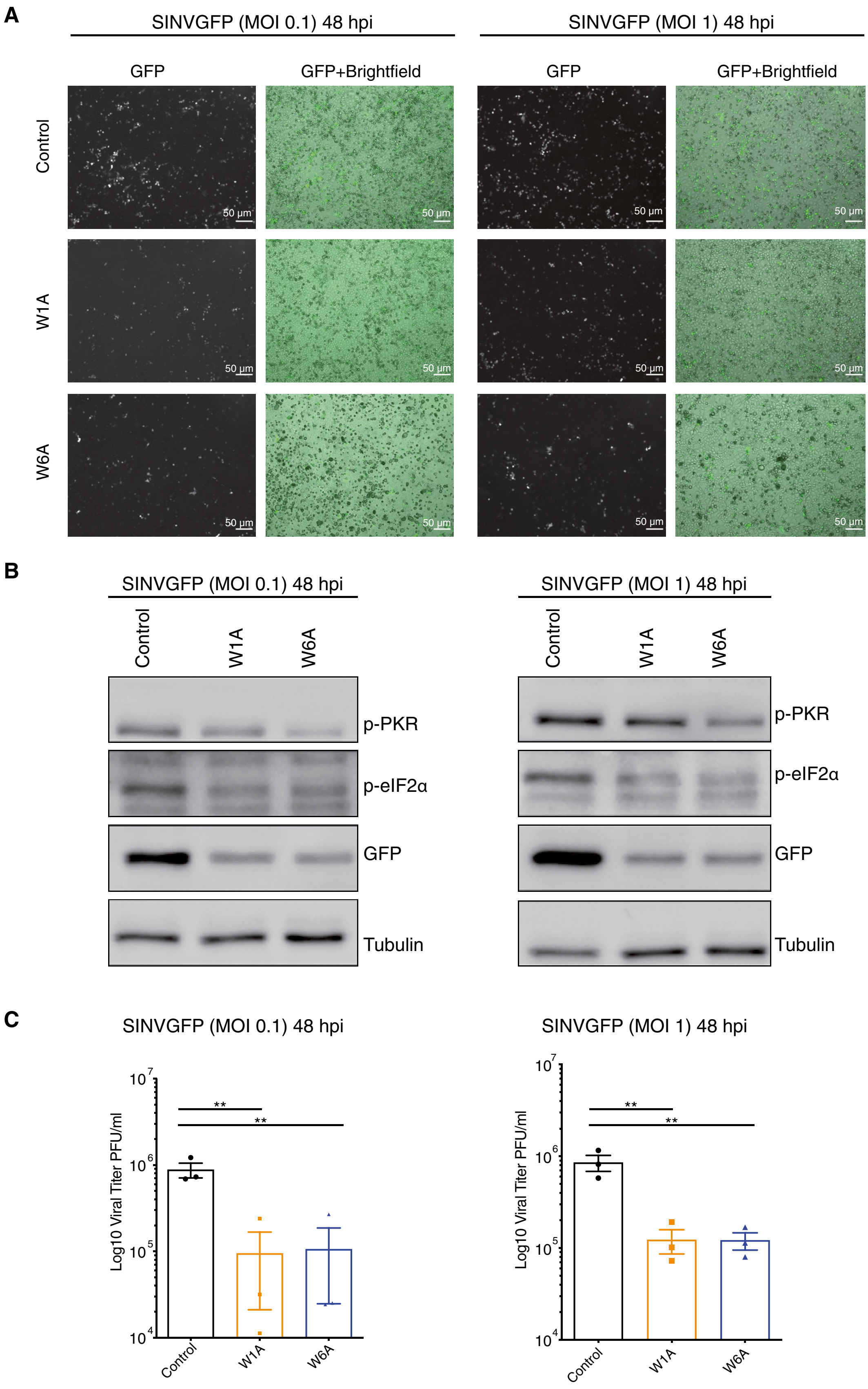
A
SINVGFP (MOI 0.1) 48 hpi
SINVGFP (MOI 1) 48 hpi
GFP
GFP+Brightfield
Control
W1A
W6A
50 µm
B
p-PKR
p-eIF2α
GFP
Tubulin
C
Log10 Viral Titer PFU/ml
10^7
10^6
10^5
10^4
**

Figure EV4.

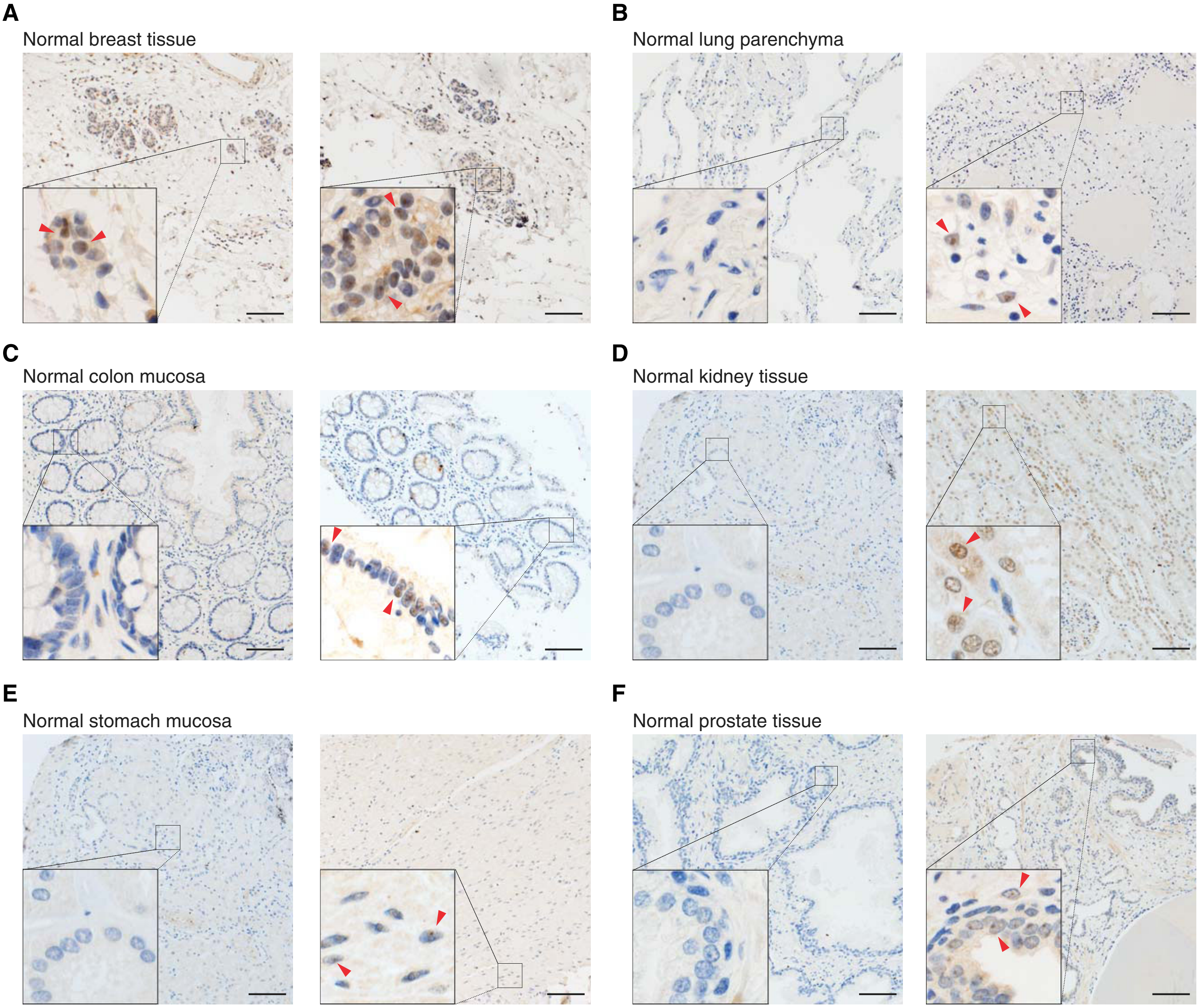

Figure EV5. Immunohistochemical analysis of AGO1x expression in non-tumoral tissues.

A–F Representative micrographs of AGO1x expression in normal breast tissue (A), lung parenchyma (B), colon mucosa (C), kidney tissue (D), stomach mucosa (E), and prostate tissue (F). Two representative non-tumoral tissue samples are shown for each organ. Insets with high magnification images highlight different degrees of positivity (red arrows) in the six different non-tumoral tissues. Scale bars, 100 µm.

 © 2020 The Authors
