# Supplementary figures and images for: Prevention of dsRNA‐induced interferon signaling by AGO1x is linked to breast cancer cell proliferation
Source: EMBO J. 2020 Aug 19;39(18):e103922. doi: 10.15252/embj.2019103922 (PMC7507497; doi:10.15252/embj.2019103922)

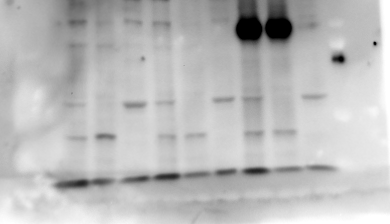

Supplement: Supplementary file 5 — Source Data for Figure 2 [file EMBJ-39-e103922-s003.zip › source_data_fig2/Fractionation of Extended Ago1 in MDA-MB-231 Cells (Blot AGO1x).tif]

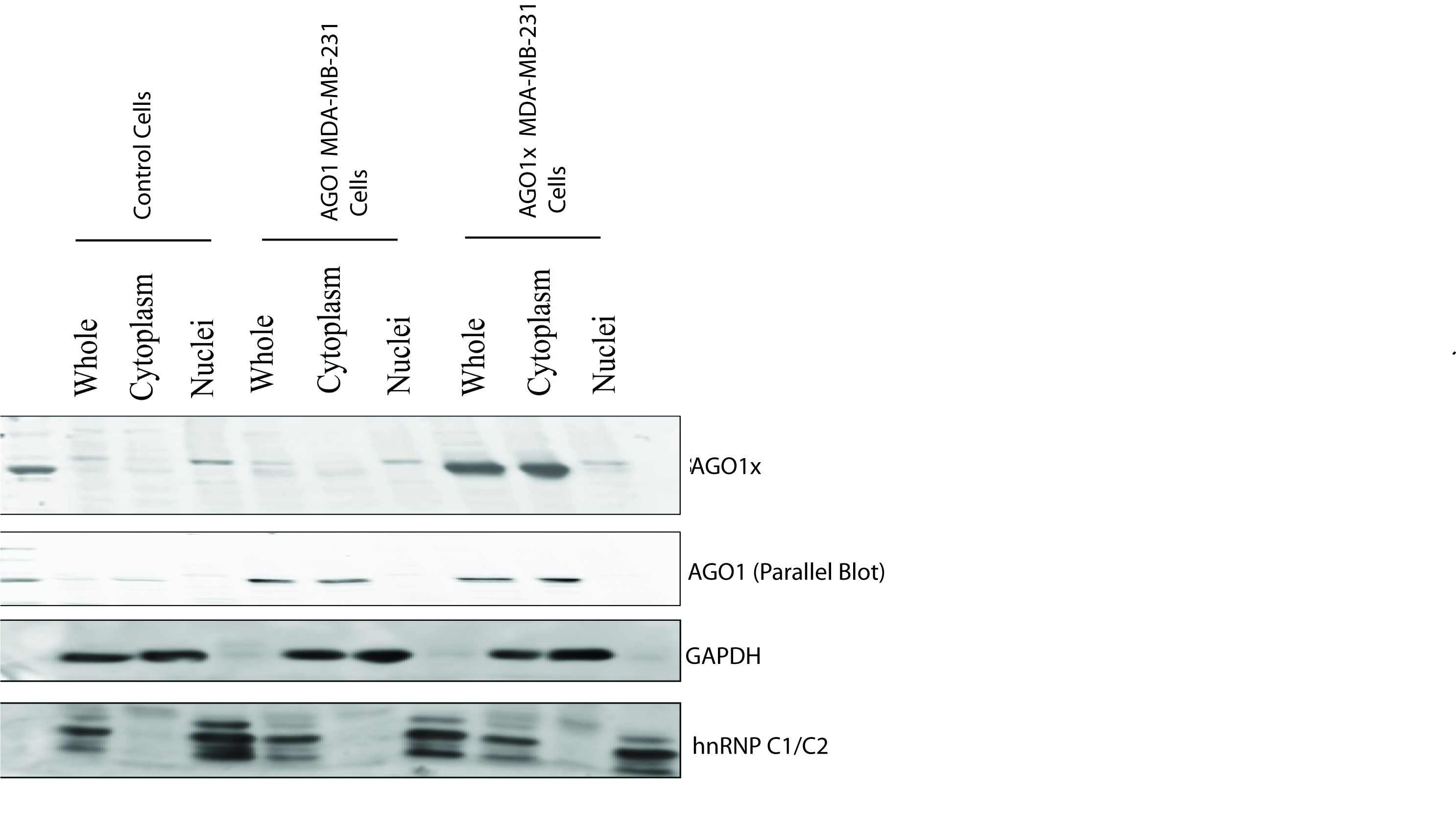

Supplement: Supplementary file 5 — Source Data for Figure 2 [file EMBJ-39-e103922-s003.zip › source_data_fig2/Fractionation Figure2C.tif]

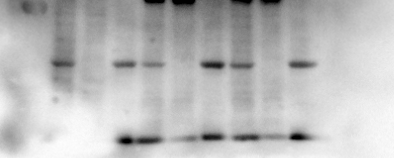

Supplement: Supplementary file 5 — Source Data for Figure 2 [file EMBJ-39-e103922-s003.zip › source_data_fig2/Fractionation of Extended Ago1 in MDA-MB-231 Cells (Blot hnRNPC1).tif]
